# Generative AI in consumer health: leveraging large language models for health literacy and clinical safety with a digital health framework

**DOI:** 10.3389/fdgth.2025.1616488

**Published:** 2025-08-26

**Authors:** Annemarie K. Tilton, Brian E. Caplan, Brian J. Cole

**Affiliations:** ^1^ Independent Researcher, Park City, UT, United States; ^2^ Department of Orthopaedics, Rush University Medical Center, Chicago, IL, United States

**Keywords:** generative AI, large language models (LLMs), consumer health education, health literacy, clinical safety, AI evaluation framework, digital health ethics

## Abstract

Generative AI, powered by large language models, is transforming consumer health by enhancing health literacy and delivering personalized health education. However, ensuring clinical safety and effectiveness requires a robust digital health framework to address risks like misinformation and inequitable communication. This mini review examines current use cases for generative AI in consumer health education, highlights persistent challenges, and proposes a clinician-informed framework to evaluate safety, usability, and effectiveness. The RECAP model—Relevance, Evidence-based, Clarity, Adaptability, and Precision—offers a pragmatic lens to guide responsible implementation of AI in patient-facing tools. By connecting insights from past digital health innovations to the opportunities and pitfalls of large language models, this paper provides both context and direction for future development.

## 1 Introduction: generative AI and digital health innovation in consumer health

A decade ago, consumer health information came from one of two sources: your doctor or a questionable corner of the internet. But increasingly, patients are turning to tools that seem to offer the best of both worlds—generative AI platforms trained on vast sets of medical data, capable of producing humanlike responses at scale. From the perspective of physicians who participate in patient care and have contributed to both clinical innovation and AI model training, this transformation is simultaneously exciting and complex.

The rise of generative AI has introduced a disruptive paradigm to healthcare communication. Generative AI refers to systems that produce original content, including text, using algorithms trained on large datasets. Large language models (LLMs) are a subset of generative AI that use probabilistic language modeling to produce human-like text. LLMs, including OpenAI's Generative Pre-trained Transformer 4 (GPT-4), Google's Med-PaLM, and Anthropic's Claude, are trained on extensive internet and medical text. They have demonstrated capabilities in summarizing medical literature, answering clinical questions, and generating patient-facing educational content (1, 2).

Such tools are being integrated into digital platforms, health systems, and patient-facing applications, and they have significant potential to bridge gaps in health literacy and expand access to evidence-based medical information (3, 4). However, concerns about misinformation, lack of contextual nuance, and patient over-reliance persist (5). Despite their prominence, few clinical frameworks exist to evaluate the appropriateness and safety of these tools from a medical perspective. Recent publications, such as Chow et al. (20), have proposed LLM evaluation frameworks focused on accuracy and tone in specific contexts like cancer care (6). However, RECAP expands this by incorporating clinical safety, adaptability, and generalizability across health domains.

This paper addresses this gap through a clinician informed framework that is grounded in health literacy, safety, and usability, offering a practical lens not often addressed in prior publications. This mini review offers a novel contribution by proposing a clinician-informed digital health evaluation framework that distinguishes it from other LLM-focused reviews through its emphasis on clinical safety, usability, and patient-centered design.

To help ground this review, sources were selected based on clinical relevance and recency (2019–2025) pertaining to the topic of AI development, digital health, and LLM use in patient-facing settings. This non-systematic approach was intended to identify key conceptual and practical insights across multiple perspectives and sources.

## 2 Applications of generative AI for health literacy in consumer health

Generative AI is already being applied across many patient-facing contexts. These tools are often hailed for their versatility, but their practical value depends on how well they function within existing healthcare communication ecosystems. The following subsections expand on both promises and pitfalls of these use cases.

### 2.1 Health literacy and generative AI chatbots

AI chatbots, software applications that use artificial intelligence to simulate human conversation, can simplify complex terminology and generate personalized responses to common health questions. This improves accessibility for users with limited medical knowledge. For example, benchmark studies like MedQA show that LLMs can perform well on structured exams, but patient-facing queries often introduce ambiguity that can reduce reliability (7). Chatbots trained without clinical oversight may overconfidently respond to symptoms without recommending appropriate follow-up (8).

### 2.2 Condition-specific content delivery

Patients managing chronic diseases such as diabetes, osteoarthritis, or breast cancer may benefit from AI-generated guidance that is tailored to their condition. However, there is wide variability in how tools handle differences in age, comorbidities, and care goals. A patient with geriatric frailty may receive advice designed for a young, active adult. Tools that fail to adjust language or urgency across subpopulations can dilute their clinical relevance (9).

### 2.3 Visit preparation and follow-up

AI tools may assist patients in preparing for a visit by helping to generate relevant questions, summarize symptoms, or clarify medical instructions. After the visit, they might reinforce medication adherence or explain discharge instructions. But if AI interpretations diverge from what clinicians intended—or use more casual or less urgent language—this may lead to confusion, conflicted messaging, or an unintended reduction in trust in the provider-patient relationship (10).

### 2.4 Mental health and lifestyle guidance

Generative models are increasingly being piloted to support cognitive-behavioral therapy (CBT) strategies, motivational interviewing prompts, and behavioral health coaching. For instance, Park et al. designed a chatbot that delivered brief motivational interviews to aid stress management. Yet, unlike structured CBT apps like Woebot or Wysa, generative models are more unpredictable and require careful oversight to avoid unintentional reinforcement of maladaptive behaviors (11–13).

### 2.5 Interactive multimedia education

Platforms like YouTube Health and patient portals may soon integrate LLMs to generate captions, voiceover explanations, or personalized summaries. While this expands accessibility, especially for users with limited literacy or disabilities, it also raises questions about narrative accuracy, cultural tone, and source transparency. Without clear attribution or a peer-review layer, misinformation can be embedded in otherwise engaging formats (2, 14).

## 3 Clinical safety challenges of large language models in consumer health

While the applications are promising, Generative AI offers no clinical assurances. Because LLMs are trained on vast datasets, their content is probabilistic and not authoritative. This leads to risks in several key areas:

### 3.1 Hallucination and inaccuracy

LLMs may fabricate references, cite non-existent studies, or assert medically unsound conclusions with unwarranted confidence (15).

### 3.2 Ambiguity and false reassurance

AI may use medically plausible language without clinical precision, omit red flag symptoms, or fail to communicate the urgency of evaluation (16).

### 3.3 Bias and representation

Biases due to unbalanced training data can lead to outputs that reflect racial, gender, or socioeconomic inequities, further marginalizing vulnerable populations (17).

### 3.4 Over-reliance and self-diagnosis

Patients may delay care, misinterpret information, or bypass provider consultation due to perceived AI authority (18).

### 3.5 Data privacy and consent

Many AI interfaces lack robust user disclosures regarding data collection, storage, and secondary use—particularly in non-clinical settings.

### 3.6 Ethical issues

Lack of transparency in how outputs are generated, unclear accountability when harm occurs, as well as in addition to the biases and privacy challenges mentioned previously contribute to a growing list of ethical concerns related to LLM use in patient-facing communication (19).

These concerns demand careful evaluation, particularly when tools are positioned for use outside of clinician oversight.

## 4 Lessons from analogous tools in digital health

Generative AI is often described as revolutionary, but many of the challenges it presents mirror earlier efforts in digital health. From symptom checkers to decision trees, healthcare has long experimented with automated tools meant to support patient understanding and behavior.

### 4.1 Legacy tools and their limitations

Early tools like WebMD, Ada Health, and Babylon offered triage assistance or self-diagnosis checklists. While helpful in some contexts, these platforms frequently delivered exhaustive lists of potential conditions with little contextual nuance. Their rigidity and lack of personalization limited their usefulness and often increased patient anxiety.

### 4.2 How generative AI differs—and doesn't

Unlike traditional rule-based tools, LLMs offer free-form, conversational responses. This opens new doors in patient engagement but introduces risks not seen in older tools—particularly hallucination, overconfidence, and context loss. LLMs can misrepresent conditions, fail to reflect urgency, or overly reassure users even when symptoms warrant escalation.

## 5 RECAP: a digital health framework for evaluating generative AI in consumer health

To guide developers, evaluators, and regulatory reviewers, the following five-point RECAP framework outlines clinician-informed criteria for consumer-facing AI health tools. Each element is rooted in experiences of patient communication and digital health challenges:

### 5.1 Relevance

Are responses specific to the user's question, contextually appropriate, and culturally sensitive? A useful tool shouldn't offer just plausible answers—it must speak to the individual patient's concern.

### 5.2 Evidence-based

Are outputs grounded in current clinical practice guidelines and appropriately cited? Without a clear foundation in evidence, AI risks becoming a digital oracle rather than a trustworthy health partner.

### 5.3 Clarity

Is language health-literate, avoiding jargon and offering accessible analogies where needed? If the message is lost in translation, it might as well not be delivered at all.

### 5.4 Adaptability

Can outputs adjust to differing levels of user education, age, or condition complexity? The best tools feel tailored, not templated.

### 5.5 Precision and safety

Does the tool recognize its limitations, defer to professional care where warranted, and flag potentially urgent issues? A tool's value is defined not only by what it says, but also by what it knows not to say.

This original digital health framework provides clinicians, developers, and platform moderators with a pragmatic tool for evaluating clinical safety and health literacy. Rather than assessing novelty alone, RECAP centers on usability, accuracy, and clinical understanding.

## 6 Applying the digital health framework for clinical safety and health literacy

The RECAP framework offers a structured lens to evaluate the quality, safety, and effectiveness of AI-generated outputs in patient-facing applications. It was developed through a combination of clinical experience, evaluation of digital health concepts, and observed challenges with current AI outputs. While inspired in part by principles from health literacy and digital health ethics, RECAP extends beyond prior models by integrating frontline clinical priorities such as contextual relevance and clinical safety, which are often missing from technical evaluation tools.

To illustrate its practical application, Table 1 evaluates sample outputs from a hypothetical chatbot responding to a basic symptom inquiry across the five RECAP domains. These examples highlight how subtle variations in language, framing, and specificity can have a meaningful impact on patient interpretation, perceived credibility, and clinical risk.

**Table 1:** Evaluating AI chatbot responses using the RECAP framework: examples of meeting and failing digital health evaluation standards.

RECAP criterion	Example output: meets standard	Example output: fails standard
Relevance	“Given your symptoms, here's tailored advice for your age and condition…”	“You may have a variety of conditions ranging from a cold to cancer”
Evidence-based	“According to CDC guidance updated in 2023…”	“People say ginger tea cures infections”
Clarity	“Your symptoms suggest you may need to see a doctor within 48 h”	“You could consider seeking professional attention soon”
Adaptability	“For someone with your condition and age, rest and hydration are especially important”	“Rest and hydration are good for anyone with these symptoms”
Precision/safety	“Your symptom combination may suggest X. Please call your doctor or visit urgent care”	“It's likely nothing serious. Wait and see”

This comparison reveals the subtle but critical distinctions in how AI-generated messages can affect patient perception and behavior. Tools that meet RECAP criteria demonstrate restraint, medical accuracy, and contextual nuance—whereas those that fail may increase risk despite sounding helpful. As LLMs are increasingly integrated into health products, RECAP can offer a shared rubric to elevate content quality and patient safety.

Relevance assesses whether AI-generated responses directly address the patient's specific question or concern in a clinically appropriate and situationally-aware manner. A relevant output demonstrates clear understanding of the patient's context, including symptoms, medical history, and stated needs, rather than offering broad, generic, or tangential information. Irrelevant responses may cause patients to be confused, delay seeking care, or lose trust in digital tools. Relevance emphasizes alignment between user intent and clinical coherence.

Evidence-based standards assess whether the AI tool's responses are grounded in current, authoritative medical guidelines or credible clinical sources. Unlike traditional web search results, generative AI models synthesize probabilistic information and can sometimes fabricate data or echo outdated practices. Tools are more trustworthy and reduce the risk of misinformation when they reference established clinical bodies, such as the CDC, WHO, or peer-reviewed guidelines. An evidence-based approach ensures that patients receive care-aligned guidance and minimizes the clinical and ethical risks of AI-mediated communication.

Clarity is essential for health literacy. Messages from the chatbot should be written in plain language, avoiding ambiguity, jargon, or vague calls to action. For example, saying “see a doctor within 48 h” provides more actionable guidance than “consider seeking professional attention soon,” which could be misinterpreted or ignored. In digital health tools, especially those used without clinician guidance, clarity directly impacts whether patients take safe, timely, and informed actions.

Adaptability reflects the AI tool's capacity to tailor communication to diverse patients with varying needs, health literacy, cultural backgrounds, and clinical complexity. For instance, a well-adapted output will deliver simplified, non-technical language for patients who are unfamiliar with medical terms and offer more detailed or nuanced guidance for those who have experience with their chronic illness. Tools that lack adaptability risk alienating patients, overwhelming them with jargon, or offering information that feels inaccessible. Effective adaptability ensures inclusivity and optimizes patient engagement across a wide range of patient demographics.

Precision and Safety refer to the AI tool's ability to deliver clinically accurate information while recognizing its own limitations. A precise response uses correct terminology, communicates appropriate urgency, and avoids overgeneralized or misleading statements. In this context, safety involves appropriately deferring to human clinicians, especially in situations that involve diagnostic uncertainty or potential risk. Importantly, LLMs must be designed to recognize red-flag symptoms, escalate when needed, and avoid implying certainty where uncertainty exists. Tools that acknowledge their limitations can help preserve clinician safety and reduce patient over-reliance.

## 7 Limitations

This review is narrative in nature and does not include any systematic search or quantitative synthesis or analysis. The RECAP framework is intended as a guiding framework for approaching the evaluation of patient-facing AI tools in consumer health. While grounded in clinical experience and literature, RECAP remains a conceptual tool that has yet to be validated through empirical studies. Future directions include structured field testing of RECAP in real-world settings, including use with AI chatbot outputs across varied health conditions and populations. This will help assess interrater consistency, practical utility, and correlation with clinical outcomes or user trust.

## 8 Conclusion

Generative AI will increasingly shape how patients seek, understand, and act on health advice. Ensuring accuracy, relevance, and safety is a shared responsibility. Clinicians must play an active role—not only to improve the quality of these tools, but to protect the patients who rely on them. With frameworks like the one proposed here, we can begin to build a more ethical, informed, and patient-centered AI future.

In doing so, we may not only enhance access to reliable health information but also restore something more essential—a balanced approach to medical information facilitating timely consensual decision-making and trust—in an era when so much of health communication feels uncertain and incomplete. Similar to how patients are guided away from online misinformation, we now have an opportunity to shape what responsible digital care will look like.
